# The Good Physician

**Published:** 1856-12

**Authors:** 


### THE GOOD PHYSICIAN.

No man can fully discharge the responsible and delicate duties of a practitioner of medicine, unless he possesses largely four cardinal qualities.

He must be Learned, Observant, Courteous and Moral.

Learned, that he may fully understand his business.

Observant, that he may daily add to his knowledge and know how to apply it.

Courteous, that he may win his way to the hearts of the suffering.

MORAL, that he may obtain and secure the highest confidence of those who place their lives in his hands.

